# In-Depth Analysis of the High Strain Rate Compressive Behavior of RTM6 Epoxy Using Digital Image Correlation

**DOI:** 10.3390/polym14091771

**Published:** 2022-04-27

**Authors:** Ahmed Elmahdy, Aldobenedetto Zotti, Anna Borriello, Mauro Zarrelli, Patricia Verleysen

**Affiliations:** 1 Materials Science and Technology-DyMaLab Research Group, Department of Electromechanical Systems and Metals Engineering, Faculty of Engineering and Architecture, Ghent University, Tech Lane Ghent Science Park, Technologiepark 46, 9052 Zwijnaarde, Belgium; patricia.verleysen@ugent.be; 2 Institute of Polymers, Composites and Biomaterials, National Research Council of Italy, P.Ie Fermi, 1, 80055 Portici, Naples, Italy; aldobenedetto.zotti@unina.it (A.Z.); borriell@unina.it (A.B.); mauro.zarrelli@cnr.it (M.Z.)

**Keywords:** RTM6 epoxy, digital image correlation, split Hopkinson bar, high strain rates, mechanical behavior

## Abstract

The aim of this paper is to study the effect of strain rate on the compressive behavior of the highly cross-linked RTM6 epoxy resin used in advanced aerospace composites. Dynamic compression tests were performed using a split Hopkinson pressure bar, along with reference quasi-static compression tests, to cover a strain rate range from 0.001 to 1035 s^−1^. Special attention was paid to the optimization of the test methodologies in order to obtain material data free of bias related to the use of different load introduction techniques and sample geometries over the considered strain rate range. In addition, the use of full-field 3D deformation measurements allowed the validation of traditional test and material assumptions. A novel self-alignment tool was developed to enable perfect interfacial contact during compression loading. The 3D digital image correlation technique was used to measure the instantaneous deformation of the sample during compression at different strain rates. Results showed a pronounced strain rate sensitivity of the RTM6 epoxy in compression. The peak yield strength increased with increasing strain rate, while the elastic modulus and Poisson’s ratio in compression were independent of the strain rate. The barreling of the sample in compression, quantified by the barreling ratio, showed an increase during the progression of the compression tests. However, the barreling ratio significantly decreased with the increasing strain rate. Finally, it was shown that neglecting the significant volume change in the yield stages gave rise to a non-negligible underestimation of the strength of the material.

## 1. Introduction

RTM6 epoxy resin is widely used as a matrix material for high-performance composites in aeronautical applications. This is mainly because of its favorable characteristics, such as high modulus and strength resulting from the high cross-linking density, low shrinkage upon curing, and good performance at elevated temperatures [1]. This type of epoxy resin is specially designed for resin transfer molding applications, which offer a low-cost alternative to conventional production processes for several primary and secondary aircraft structures such as fairings, stabilizers, engine fan blades, wing ribs, and spars [2]. However, these structures are typically subjected to catastrophic impact loads such as bird and hail strikes. Therefore, in order to effectively design these structures for impact loads, it is important to understand the behavior of the materials at high strain rates.

Several researchers studied the high strain rate behavior of several similar epoxy resins. Gomez-del Rio et al. [3] reported an increase in elastic modulus and yield strength of a bisphenol A diglycidyl ether (DGEBA) epoxy with increasing strain rates from 0.0025 to 2500 s^−1^ using the split Hopkinson pressure bar (SHPB) technique. Fard et al. [4] and Littell et al. [5] investigated the mechanical behavior of Epon E 863 and Epon 862, respectively, at quasi-static strain rates ranging from 10^−5^ to 10^−1^ s^−1^. Results showed an increase in tensile and compression strengths and elastic modulus and a decrease in fracture strains with the increase in strain rate. Bernard et al. [6] studied the effect of strain rate on the compressive behavior of a laboratory prepared bisphenol A diglycidyl ether (DGEBA) epoxy at strain rates up to 1770 s^−1^ using an SHPB setup. Results also showed a strong strain rate dependency of the elastic modulus and the yield strength, however, and contrary to the findings of Gomez-del Rio [3] and Poulain et al. [7], no strain-softening was observed. Naik et al. [8] also reported an increase in compressive strength with increasing strain rates of LY556 epoxy using an SHPB setup at strain rates up to 1890 s^−1^.

While the previous studies focused mainly on light and medium crosslinked epoxy systems, very few studies are available for the high strain rate behavior of highly cross-linked epoxy systems such as RTM6. Gerlach et al. [9] showed an increase in compressive elastic modulus and yield strength of RTM6 epoxy with increasing strain rates from 10^−3^ up to 10^4^ s^−1^. However, the strains obtained in the quasi-static regime were based on the cross-head displacement of the testing machine and were not corrected for the machine compliance, therefore, the elastic modulus was only reported as an apparent value. Morelle et al. [10] showed that in the quasi-static regime, the elastic modulus in compression was nearly independent of the strain rate, however, the compressive yield strength increased with increasing the strain rates.

In addition to the lack of studies for the highly cross-linked epoxy systems at high strain rates, there are several challenges related to compression testing of polymers using high strain rate testing, including SHPB techniques. These challenges are mainly: the design of sample geometry, the boundary conditions at the loading interfaces, and assumptions related to the material itself.

The design of a compressive sample geometry is a crucial aspect in determining the correct stress–strain response of polymers. This becomes even more significant at a high strain rate, due to waves propagating inside the sample material. As such, several additional, interconnected requirements have to be achieved:

1. The compression specimen should be short enough to achieve an early quasi-static stress equilibrium and to increase the achievable maximum strain rate, yet the sample length should be long enough to minimize the influence from the loading interfaces;
2. A state of uniform stress and strain has to be achieved in the sample;
3. The design of the compression specimen should prevent failure due to buckling and should reduce inertia effects during dynamic compression.

Several studies were performed to determine the most suitable height to diameter ratio for cylindrical samples to achieve the abovementioned requirements [11,12,13,14]. A height to diameter ratio of 1 is most often recommended to reduce the barreling effect due to interfacial friction [13]. However, this height to diameter ratio might not be ideal for high strain rate compression tests, due to the additional requirements of quasi-static equilibrium and reduction of inertia effects [15]. This becomes more significant with polymers having low wave propagation speeds [16]. Therefore, a height to diameter ratio of 0.5 is usually applied in compression testing of polymers at high strain rates [3,9,15]. In this case, however, interfacial friction effects and barreling of the sample become important. Interfacial friction can increase the apparent strength of tested polymer due to boundary confinement, in addition to giving rise to a non-uniform stress state within the sample, as shown by Morelle et al. [10] and Zhong et al. [14]. Therefore, sample design, combined with monitoring and accounting for barreling, is important. The Digital Image Correlation (DIC) technique has proven to be very useful in studying the interfacial friction effect in compression testing [17]. DIC can provide the full 3D displacement fields, and is free from the errors which are typically associated with other techniques, such as linear variable displacements transducers (LVDTs) when measuring the radial deformation of compression samples [11].

In addition to the size and geometry of the sample, the boundary conditions and load introduction to the sample interfaces can significantly affect the final measured stress–strain curve and the validity of the SHPB test assumptions. High-strength sample materials can indent the more compliant bars, which causes non-flat loading interfaces and radial confinement of the sample. Both will lead to inaccuracies and even errors in stress and strain measurements [18]. A typical solution is the addition of high-strength, hard metallic, or ceramic inserts at both bar interfaces. However, care should be taken to design such inserts without introducing additional mechanical impedance mismatch and disturbing the propagation of the waves [18,19]. The lack of parallelism between loading interfaces can induce either an irregular stress–strain response at low strain levels or induce premature failure due to bending [11]. In addition, the lack of parallelism between the loading interfaces can result in tilting of the sample during loading, which can introduce errors in the determination of the dynamic elastic modulus in compression [20]. Spherical joints have been introduced to provide self-alignment capabilities at high strain rates [21]. However, the design of these spherical joints is susceptible to instabilities, especially if the centerlines of the sample and the bars are not perfectly aligned. Therefore, new design concepts for self-alignment attachment are required to provide both good contact conditions and good stability during dynamic testing.

As in the case of metals, conservation of volume in polymers is very often assumed during yielding and post-yielding stages of deformation [3,6,7,9,12]. However, seeing the differences in deformation mechanisms, which involve molecular restructuring and entanglement of polymer chains, the assumption is less obvious for polymers. Indeed, it was shown by Jerabek et al. [11] that post-yielding behavior at quasi-static strain rates can involve either an increase or a decrease in volume. This indicates that the assumption of volume conservation during all stages of deformation should be validated, including at high strain rates, considering the strain rate sensitivity of polymers [22].

The aim of this paper is to provide a detailed analysis and study of the compressive behavior of the highly crosslinked RTM6 epoxy at high strain rates. High strain rate tests were performed using the SHPB technique. Reference quasi-static tests were also performed to study the compressive behavior of RTM6 at a wide range of strain rates. Special attention was paid to extending the capabilities of the SHPB technique in order to address the previously mentioned testing challenges, by incorporating a novel self-aligning specimen interface. Additionally, the 3D DIC technique was used to accurately measure the elastic properties and the yield strength of the epoxy at high strain rates, in order to study the effects of interfacial friction and sample barreling and to assess the assumptions of volume conservation. Reliable data for the effect of strain rate on the elastic modulus, the Poisson’s ratio, and the peak yield strength in compression are presented and discussed.

## 2. Materials and Methods

### 2.1. Specimen Materials and Geometry

**Figure 1:**
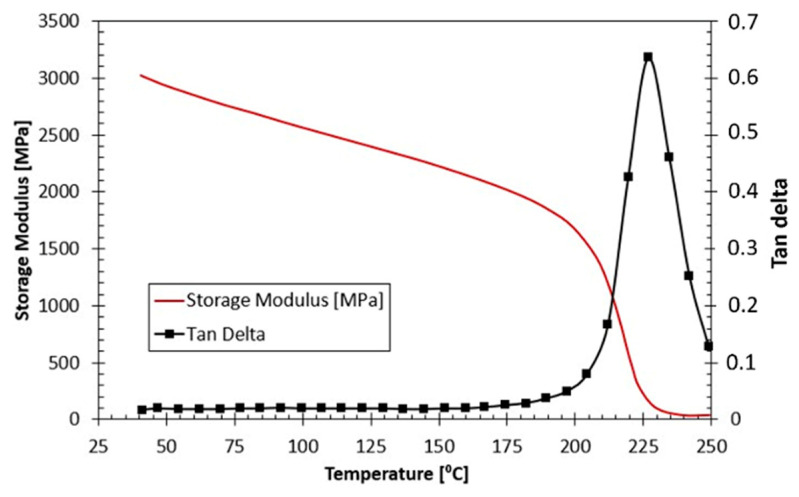
DMA results, that is, tan delta and storage modulus, of the RTM6 epoxy resin.

The epoxy resin used in this study was RTM6 (supplied by Hexcel Composites, Duxford, UK). It is a monocomponent system based on a tetra-functional tetraglycidyl methylene dianiline (TGMDA) as epoxy resin and 4,4′-methylenebis (2,6-diethylaniline)/4,4′-methylenebis (2-isopropyl-6-methylaniline) as hardeners. RTM6 was characterized by an equivalent weight of 116 g/eq and a viscosity of 33 mPa·s at 120 °C. The resin was degassed in a vacuum oven (SALVIS VC20, Rotkreuz, Switzerland) at 90 °C for 30 min, and then poured into a cylindrical steel mold and oven cured according to the manufacturer’s temperature profile (160 °C for 90 min, followed by 180 °C for 2 h). Finally, the epoxy resin cylinder was left in the oven for 24 h to cool to room temperature. Figure 1 shows the Dynamic Mechanical Analysis (DMA) results of the cured resin obtained using a DMA Q800 system (TA Instruments, Milan, Italy) operating in a double cantilever configuration. The glass transition temperature, which corresponds to the peak of the tan-delta curve, was 226.6 ± 0.2 °C.

**Figure 2:**
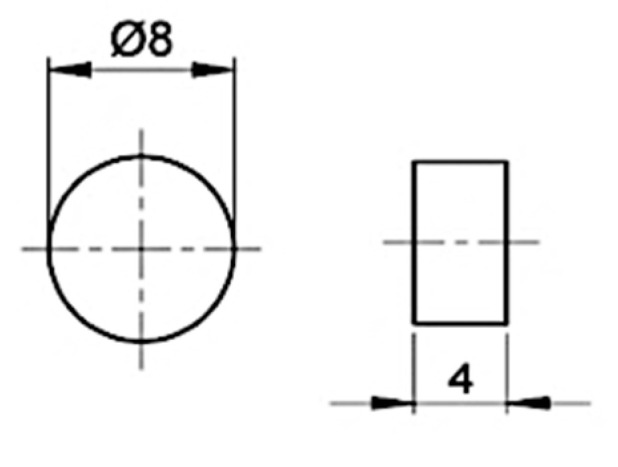
Dimensions of the cylindrical compression sample.

The cured cylindrical rods of the resin were cut into disk-shaped samples, with 8 mm diameter and 4 mm height (i.e., height to diameter ratio is 0.5), as shown in Figure 2. Identical samples and loading conditions were used for both reference quasi-static and high strain rate tests, in order to eliminate any discrepancies related to sample geometry or size or testing boundary conditions.

### 2.2. Quasi-Static Testing

Quasi-static tests were performed using a universal testing machine (Instron 5569, supplied by Instron, Boechout, Belgium) equipped with a 50 kN load cell. Crosshead testing speeds were 0.2, 2, and 20 mm/min, corresponding to strain rates of 0.001, 0.01, and 0.1 s^−1^, respectively, in the samples. Samples were placed between two steel bars, where the upper bar was directly connected to the load cell, while the lower bar was directly connected to the base of the testing machine.

**Figure 3:**
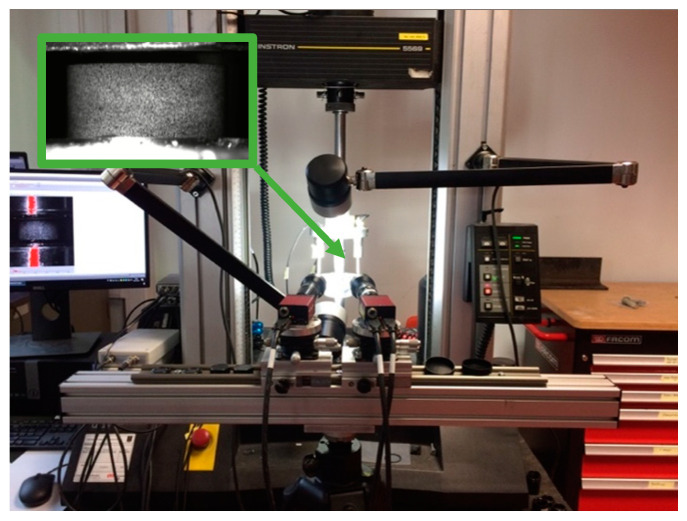
Quasi-static compression setup with magnified speckled sample (top left).

Displacements and strains in the sample were measured using 3 LVDTs (supplied by RDP Group, Le Spijkenisse, The Netherlands) and fixed on the steel bars. The displacements obtained from the LVDTs were corrected for bar compliance during compression. Additionally, a low-speed 3D DIC setup was used to locally measure the full-field displacements and strains on the sample surface. The optical setup consisted of two 5-megapixel machine vision cameras (stingray 504b by Allied Vision, Stadtroda, Germany) positioned under a stereo angle of 18.85°. Each camera was equipped with two lenses of 100 mm fixed focal length (Edmund Optics, York, UK). A black-on-white speckle pattern was applied to the surface of the samples prior to testing. During deformation, images of the speckled samples were recorded at a resolution of 2452 × 2056 pixels^2^. The average speckle size of the samples tested at quasi-static conditions was approx. 0.09 mm, which corresponds to 10 pixels on the images. In order to determine the intrinsic and extrinsic parameters of the 3D DIC system, a calibration was carried out using a small etched glass calibration grid, having 9 × 9 dots and a pitch of 1.34 mm between the centers of the dots. Figure 3 shows the quasi-static setup used. The average displacement resolutions were approx. 0.05 and 5 μm for in-plane and out-of-plane displacements, respectively, for the quasi-static 3D DIC system, and 0.18 μm for the LVDTs.

### 2.3. High Strain Rate Testing

**Figure 4:**
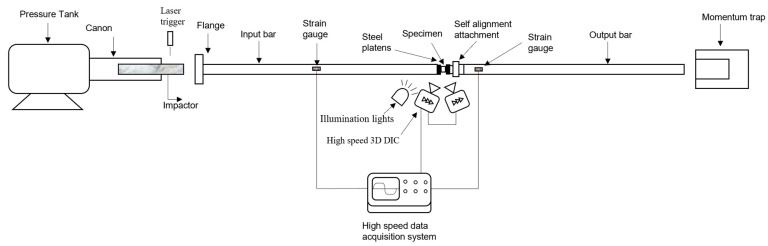
Schematic of the SHPB setup.

The high strain rate compression experiments were performed using the split Hopkinson pressure bar facility at Ghent University. The details of the setup were explained in previous work [23,24]. Figure 4 shows a schematic of the SHPB setup. The cylindrical sample was placed between two bars (input and output bars) made of aluminum and having lengths of 6 and 3 m, respectively. When an impactor was accelerated towards the incident bar, a dynamic incident compressive loading wave was generated. When this incident compressive wave reached the sample, a part of the incident wave was reflected back to the input bar, and a part was transmitted to the output bar. The time histories of the incident $\varepsilon_{i}$, the reflected $\varepsilon_{r}$, and the transmitted $\varepsilon_{t}$ strain waves were measured using strain gauges attached on the bars. The measured strain waves were time shifted from the location of the strain gauges to the specimen/bar interfaces [18]. In addition, wave dispersion effects were eliminated by using a polymeric impactor as an integrated pulse shaper. The impactor was accelerated with velocities of 8, 11, and 14 m/s. By achieving a state of quasi-static force equilibrium, and neglecting the inertia effects [9,15], the one-dimensional wave propagation analysis—developed by Kolsky [25]—can be used to calculate the time histories of the average axial strain rate ${\dot{\varepsilon }}^{Hop}$, strain $\varepsilon^{Hop}$, and stress $\sigma^{Hop}$ in the sample as follows:

(1) $${\dot{\varepsilon }}^{Hop}=-2\frac{C_{o}}{H_{s}}\varepsilon_{r}\left(t\right)$$

(2) $$\varepsilon^{Hop}=-2\frac{C_{o}}{H_{s}}\int_{0}^{t}\varepsilon_{r}\left(t\right)dt$$

(3) $$\sigma^{Hop}=E_{b}\frac{A_{b}}{A_{s}}\varepsilon_{t}$$

where $C_{0}$ is the elastic wave speed in the bar material, $H_{s}$ is the height of the sample, $E_{b}$ is the elastic modulus of the bar material, $A_{b}$ and $A_{s}$ are the cross-sectional areas of the bar and the sample, respectively.

**Figure 5:**
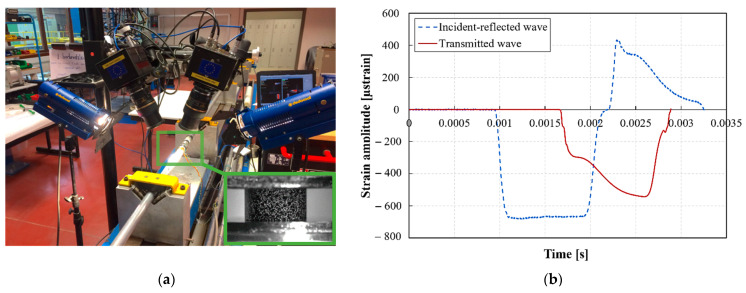
(**a**) High-speed 3D DIC setup used, with detail of the speckled sample (bottom right), (**b**) Example of the incident, the reflected, and the transmitted wave recorded on the Hopkinson bars during a dynamic compression experiment.

The local strains and strain rates on the surface of the sample were measured using the high-speed 3D DIC technique. The optical system consisted of two Photron Mini AX200 high-speed cameras (supplied by Photron, Brussels, Belgium) positioned at a stereo angle of 26.36° and equipped with two lenses having a 90 mm fixed focal length. A black-on-white speckle pattern was applied to the surface of the samples prior to testing. Images were recorded at a resolution of 384 × 265 pixels^2^ and a rate of 54,000 images/s. In order to determine the intrinsic and extrinsic parameters of the DIC system, similar to the 3D DIC system of the quasi-static setup, a calibration was carried out using a small etched glass calibration grid, having 9 × 9 dots and a pitch of 1.78 mm between the centers of the dots. The exposure signal of the camera sensors was synchronized with the measured strain signals on the bars using the same high-speed data acquisition system used to measure the strains on the bars. The average speckle size of the dynamically tested samples was approx. 0.16 mm, corresponding to three pixels on the images. The average displacement resolutions were approx. 0.7 and 2 μm for in-plane and out-of-plane displacements, respectively. The better out-of-plane displacement resolution compared to the quasi-static optical system resolution is related to the use of a slightly larger stereo angle in the high-speed 3D DIC setup. Figure 5 shows the high-speed 3D DIC system used, and an example of the incident, the reflected, and the transmitted waves recorded in one of the dynamic compression experiments.

### 2.4. Sample Fixing and Alignment

**Figure 6:**
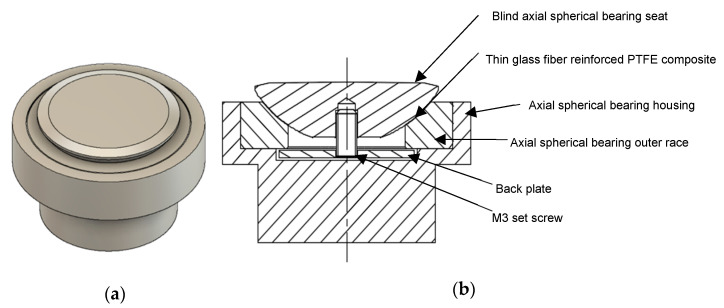
Self-alignment attachment for the compression tests: (**a**) isometric view, (**b**) and section front view.

A novel self-alignment attachment was developed for the quasi-static and high strain rate compression tests, as shown in Figure 6. This attachment was developed in order to provide full contact between the loading surface of the bars and the specimen interfaces even if the specimen interfaces lack perfect parallelism. The attachment is based on a modified axial spherical plain bearing (INA GE30-AX), which was modified by changing its steel inner race to a blind spherical seat for the compression sample. The seat was cut from a metallic ball and matched the spherical cavity of the bearing’s outer race. The contact surface between the spherical seat and the bearing’s outer race was made of a thin layer of glass fiber reinforced PTFE composite to minimize friction. The seat and the outer race were kept in contact by means of a backplate and a small set screw. This prevents the spherical seat from falling off during loading. The contact surface between the backplate and the outer race was greased before assembly to minimize friction. The outer race was press-fitted into a metallic housing, in which sufficient clearances were considered to allow the spherical seat to move and accommodate the sample. For the high strain rate tests, the housing and the spherical seat were attached to the output bar. Additionally, they were made from aluminum to match the material of the output bar and minimize the impedance mismatch. Due to the unavoidable difference in the cross-sectional area in the housing and the different materials used, some small wave reflections were expected due to impedance mismatch. However, as shown in Figure 5b, only very small, negligible oscillations were observed in the transmitted signal. A thin steel platen was attached at the loading interface with the sample to avoid indentations in the aluminum seat. For the quasi-static testing, the same self-alignment attachment configuration was used, however, the parts made of aluminum were replaced by steel parts. For both the quasi-static and high strain rate tests, and to reduce barreling and interfacial friction, the loading interfaces with the sample were polished to a mirror finish, and carefully lubricated with a PTFE lubricant. Moreover, the centerlines of the samples and the bars were carefully aligned with a special alignment tool.

### 2.5. DIC Data Reduction and Processing Parameters

The recorded images of the deforming samples during the tests were processed and analyzed using MatchID commercial digital image correlation software (supplied by MatchID, Ghent, Belgium). The processing parameters used for both quasi-static and dynamic tests are summarized in Table 1. Using these parameters, it was possible to achieve a strain resolution of approx. 155 microstrains in quasi-static tests and approx. 400 microstrains in high strain rate experiments. Since the shape of the sample is cylindrical, only the central part of the sample lies in the cameras’ focal plane. Therefore, at each moment during the quasi-static and dynamic tests, the average full-field in-plane strains and out-of-plane displacements were extracted in an area of 3.5 × 3.5 mm^2^ at the center of the sample. The axial engineering strain $\varepsilon^{DIC}$ was calculated based on the reference Biot strain convention, while the axial true strain $\varepsilon_{t}^{DIC}$ was calculated based on the Hencky strain convention.

In addition to the axial strain, DIC processing also allowed to obtain the transverse component of the strain $\varepsilon_{hoop}^{DIC}$ (also denoted as hoop strain). The hoop strain was calculated using the radial displacement $u_{r}$ obtained from the DIC measurements and the initial radius $r_{0}$ as follows:

(4) $$\varepsilon_{hoop}^{DIC}=\frac{u_{r}}{r_{o}}$$

True stresses are commonly calculated from engineering stresses assuming conservation of volume during the deformation process. Using the DIC strain and engineering stress obtained by Equation (3), the true stress based on DIC strains and conservation of volume $\sigma_{t}^{DIC,Vol}$ can be calculated using the following relation:

(5) $$\sigma_{t}^{DIC,Vol}=\sigma^{Hop}\cdot \left(1+\varepsilon^{DIC}\right)$$

While the assumption of the conservation of volume might be a valid assumption for the plastic deformation of metals, it might not be the case for polymers. Since the hoop strain was also measured in this study, the assumption of volume conservation here becomes obsolete and the true stresses can be calculated based on the instantaneous cross-sectional area using the following relation:

(6) $$\sigma_{t}^{DIC}=\frac{F}{A}=\frac{F}{\pi r^{2}}=\frac{F}{\pi r_{0}^{2}{\left(1+\varepsilon_{hoop}^{DIC}\right)}^{2}}=\frac{\sigma^{Hop}}{{\left(1+\varepsilon_{hoop}^{DIC}\right)}^{2}}$$

In order to further experimentally assess the assumption of conservation of volume, the total volumetric strain $\varepsilon_{V}$ can be determined based on the ratio between the final volume $V_{f}$ and the initial volume $V_{0}$ of the sample using the following relation:

(7) $$\varepsilon_{V}=\frac{V_{f}}{V_{o}}-1=\left[{\left(1+\varepsilon_{hoop}^{DIC}\right)}^{2}\cdot \left(1+\varepsilon^{DIC}\right)\right]-1$$

The total volumetric strain includes both the elastic volumetric strain $\varepsilon_{V}^{ela}$ and the non-elastic volumetric strain $\varepsilon_{V}^{non-ela}$. The non-elastic volumetric strain can be determined using the following relation:

(8) $$\varepsilon_{V}^{non-ela}=\varepsilon_{V}-\varepsilon_{V}^{ela}=\left[\left[{\left(1+\varepsilon_{hoop}^{DIC}\right)}^{2}\cdot \left(1+\varepsilon^{DIC}\right)\right]-1\right]-\left[\left(1-2\nu \right)\cdot \frac{\sigma_{t}^{DIC}}{E}\right]$$

where E is the elastic modulus and ν is the Poisson’s ratio calculated in the linear-elastic region at the early stage of loading using the following relation:

(9) $$\nu =-\frac{\varepsilon_{hoop}^{DIC}}{\varepsilon^{DIC}}$$

In order to assess the validity of the classical Hopkinson stress and strain calculation using Equations (2) and (3), the axial compressive true stress $\sigma_{t}^{Hop,vol}$, assuming conservation of volume, was calculated from the stress obtained by Equation (3):

(10) $$\sigma_{t}^{Hop,Vol}=\sigma^{Hop}\cdot \left(1+\varepsilon^{Hop}\right)$$

The true compression strain can be calculated from Equation (2) using:

(11) $$\varepsilon_{t}^{Hop}=\ln\left(1+\varepsilon^{Hop}\right)$$

## 3. Results and Discussion

### 3.1. Quasi-Static Force Equilibrium and Strain Rate Evolution in the SHPB Tests

**Figure 7:**
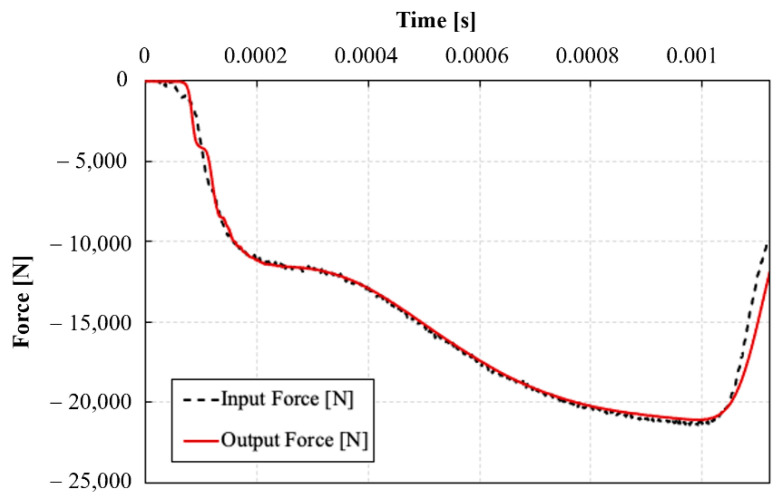
Comparison between the time histories of the forces at the specimen/bar interfaces during an SHPB test.

Figure 7 represents a comparison between the time histories of the forces at the input and output bar/specimen interfaces during one of the dynamic compression experiments. It can be seen that the input bar force signal showed an excellent agreement with the output bar force signal starting from approximately 100 µs. This indicates the achievement of a quasi-static force, and hence stress, equilibrium in the sample from the early stages of deformation. An early quasi-static equilibrium was achieved for all dynamically tested specimens. It should be noted that the oscillations resulting from the self-alignment attachment on the output bar were small in magnitude and occurred at stress levels in the sample below 100 MPa, which is well into the elastic stage [10].

**Figure 8:**
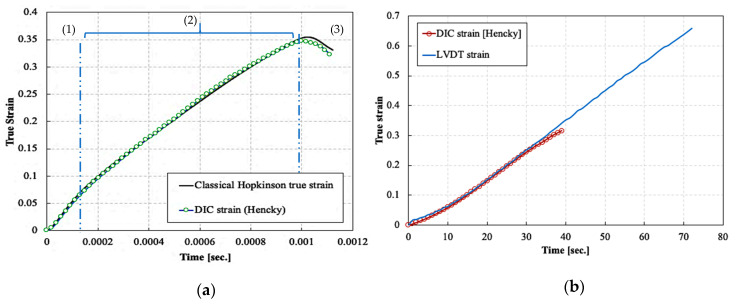
Strain-time history: (**a**) during a high strain rate compression experiment, (**b**) during a quasi-static strain compression experiment.

Figure 8a represents the true axial strain time history of one of the high strain rate compression experiments using the classical Hopkinson analysis Equation (11) and the true DIC axial strain. An excellent agreement was obtained between DIC and Hopkinson axial strains. The speckle pattern could not follow the deformation of the samples beyond approx. 35% true strain. Therefore, DIC strains were not reported beyond that limit. In the dynamic tests, the samples were deformed in approximately 1 ms. The progression of the strain with respect to time consisted mainly of three stages: (1) an initial stage with times ranging from 0 to ~100 μs, which included the establishment of the quasi-static stress equilibrium followed by the elastic response of the material, (2) a second stage in which a constant strain rate was established, starting from time ~100 μs and up to 1 ms, and (3) a third stage, after 1 ms, in which the strain decreased correspondingly to the unloading of the sample. None of the dynamically tested samples failed during the compression tests, and some elastic springback was observed in all samples. Consequently, the maximum strain reached in the dynamic tests cannot be considered the failure strain. For all dynamic tests, the average true strain rates corresponding to the elastic constants (i.e., elastic modulus and Poisson’s ratio) were calculated in the first stage, while the average true strain rates corresponding to yielding were calculated in the second stage. These average true strain rates were calculated as the slope of the true strain-time curve in the respective stages. The average strain rates achieved for all the dynamic compression tests were in the range of 285 to 1094 s^−1^.

Figure 8b shows the true strain time history of a quasi-static compression experiment, using the LVDTs and DIC. The true strains measured using the LVDTs were corrected for the bar compliance, which is the compliance resulting from the compression of the portion of the bars contained within the LVDTs measurement section and the self-alignment device. The quasi-statically tested samples were compressed to failure. Similar to the high strain rate case, the true strains measured using the DIC technique were in excellent agreement with the strains measured using the LVDTs. Also for the static tests, the speckle pattern could not follow the deformation of the samples beyond approx. 30% true strain.

### 3.2. Interfacial Friction and Sample Barreling

**Figure 9:**
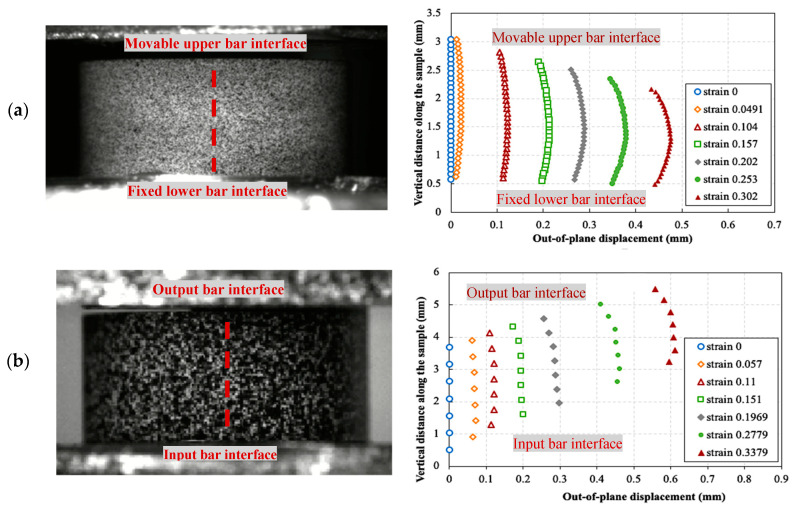
Out-of-plane (radial) displacement and vertical position of the vertical center line on the sample surface indicated in red at different levels of true axial strain: (**a**) during a quasi-static test, (**b**) during a high strain rate test.

As mentioned earlier, reducing the interfacial friction to limit sample barreling is crucial to ensure homogenous stresses and strains within the compression sample. Using the 3D DIC technique, it was possible to monitor the radial displacement during the deformation of the sample and thus directly measure the barreling at the surface of the sample. This was performed by extracting the radial displacements on fixed several points along the vertical center line on the surface of the sample during the deformation. Figure 9 shows examples of the extracted radial (or out-of-plane) displacements for a quasi-static test and a high strain rate test at different levels of true axial strain. The upward movement of the curves, which is observed in the dynamic tests, is due to the movement of the input and the output bars. From a qualitative point of view, it can be seen that the barreling was negligible up to 0.1 true axial strain for both tests, showing a nearly straight contour of the sample. The barreling was quantified by calculating the barreling ratio, defined as $\left(\Delta t_{b}/r_{o}\right)$, where $\Delta t_{b}$ is the difference between the radial displacements in the middle of the sample and the most confined point close to one of the loading interfaces, and $r_{o}$ is the initial radius of the sample.

**Figure 10:**
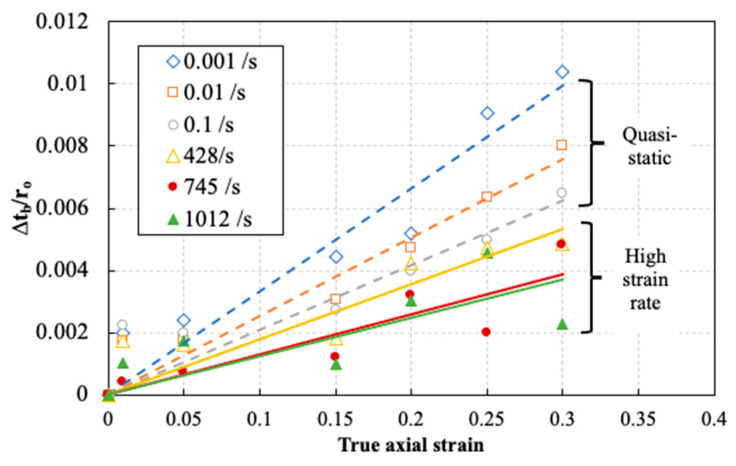
Evolution of the barreling ratio during compression tests at different strain rates.

Figure 10 shows an example of the evolution of the barreling ratio as a function of true axial strain for several representative static and dynamic tests. The solid lines indicate a linear least square fit, with R^2^ values higher than 82%. The indicated strain rates correspond to the strain rate reached at the yielding stage. It can be seen that the barreling ratio increased during the progression of the compression tests. However, the barreling significantly decreased with increasing strain rate. Indeed, at a true axial strain of 0.3, the barreling ratio of approx. 1% obtained in the sample tested at the lowest strain rate, that is, 0.001 s^−1^, was reduced with a factor of approx. 5 to 0.2% in the sample tested at a strain rate of 1012 s^−1^. The overall low barreling ratios indicate good lubrication conditions with minimum barreling up to 0.3 true axial strain in all compression tests.

### 3.3. Axial Strain Homogeneity and Conservation of Volume

**Figure 11:**
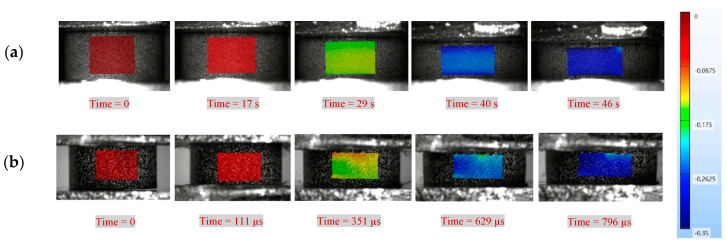
Evolution of axial true strain fields during compression testing: (**a**) a quasi-static test at 0.008 s^−1^, (**b**) a high strain rate test at 428 s^−1^.

Figure 11 shows the evolution of the true axial compressive strain fields during a quasi-static experiment at a strain rate of 0.01 s^−1^ and a dynamic experiment with a strain rate of 428 s^−1^ at the yielding stage. The images were recorded during the deformation from time 0 to 46 s for the quasi-static experiment, and 0 to 796 µs for the high strain rate experiment. The color map indicates the strain levels measured on the surface of the sample, blue corresponds to the highest true strain of 0.35 and red to a true strain of 0. It can be seen that homogeneous strain fields developed at both the quasi-static and the high strain rate.

**Figure 12:**
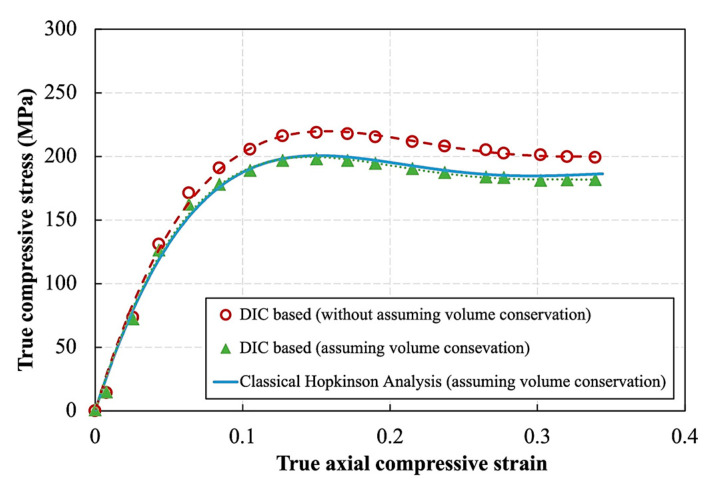
True stress–true strain curve of a high strain rate compression experiment on the epoxy resin.

Figure 12 shows a comparison between the true stress–true strain curves of an SHPB experiment obtained using three different formulations of stress and strain: one fully based on the classical Hopkinson analysis (Equations (10) and (11)), one substituting the Hopkinson axial strain by DIC strain data Equation (5)), and one based on DIC measurement of the actual cross section (Equation (6)). The first two formulations assume volume conservation, and they quasi coincide. However, both curves differ from the curve based on the direct measurement of DIC, which does not require volume conservation. The latter shows higher stress levels starting from a true strain of 0.05, reaching approx. 20 MPa or 10% difference at a true strain of 0.15. The difference between the curves gives the first indication that the volume might not be valid during the test.

**Figure 13:**
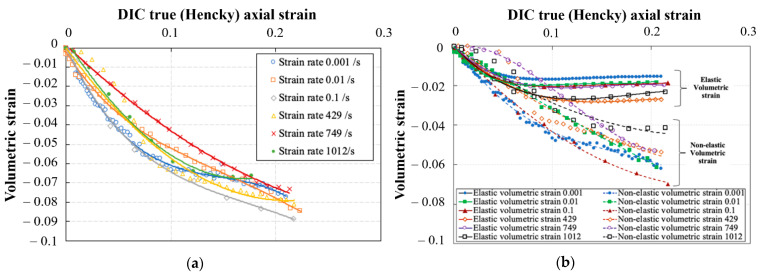
Variation of the relative volume change on the neat epoxy resin as a function of true axial strains at different strain rates during compression: (**a**) volumetric strain, (**b**) elastic and non-elastic volumetric strain.

Figure 13a shows representative curves for the total volumetric strain (Equation (7)) obtained for quasi-static and high strain rate tests up to 0.2 axial strain. The indicated strain rates correspond to the strain rates at the yielding stage. As expected, the volume decreases as a function of imposed strain, for all strain rates. The reduction in volume is attributed to the compaction of the material as a result of the compressive hydrostatic pressure. The volume of the sample is reduced by approx. 3% to 6% at an axial strain of 0.07, which is roughly the average strain level at which the yielding of the material starts. At true axial strains higher than 0.07, the volume reduction rate is slightly reduced. The presence of this transition point indicates that the reduction in volume varies in the elastic and non-elastic stages of loading. Additionally, the strain rate does not have a significant effect on volume reduction. Figure 13b shows the elastic and non-elastic volumetric strains (Equation (8)) up to 0.2 axial strain. Both the elastic and non-elastic volumetric strains follow a similar trend in volume reduction up to strains approx. 0.07. Beyond axial strain of 0.07, the elastic volumetric strains show nearly plateauing behavior up to values of 1.5% to 2.5% of the total volume at all strain rates. Whereas the non-elastic volumetric strains show and increase to reach a maximum ranging between 4.5% to 7% of the total volume at all strain rates. The effect of strain rate on both the elastic and non-elastic volumetric strains is also not significant. The volumetric strains indicate that in the yield stage, and contrary to what is often assumed, a permanent volume change occurs. Similar results were reported by Jerabek et al. [11] for polypropylene in compression at quasi-static strain rates. However, in the yielding stage, the authors indicated that the material could experience either an increase or a decrease in the volume depending on the position at which the Poisson’s ratio was measured on the sample, and the stage at which the Poisson’s ratio was calculated. In the present study, the Poisson’s ratio used in Equation (8) was measured in the center of the sample, and extracted from the initial linear part of the stress–strain curve. Indeed, as evident from the DMA results in Figure 1, the tan delta—which is a direct measure of the viscous component of the material behavior—is nearly zero in the temperature range of 23 °C up to 150 °C. Additionally, as will be shown in Section 3.5, the elastic modulus and the Poisson’s ratio calculated in the linear part only seemed to be marginally affected by the strain rate. It is therefore assumed that the material response at the low deformation range is dominantly elastic rather than viscoelastic. Based on the previous analysis, the peak yield strength reported later in Section 3.4 is based on the stress–strain curves without the conservation of volume.

### 3.4. Compressive Stress–Strain Response of RTM6 Epoxy at Different Strain Rates

**Figure 14:**
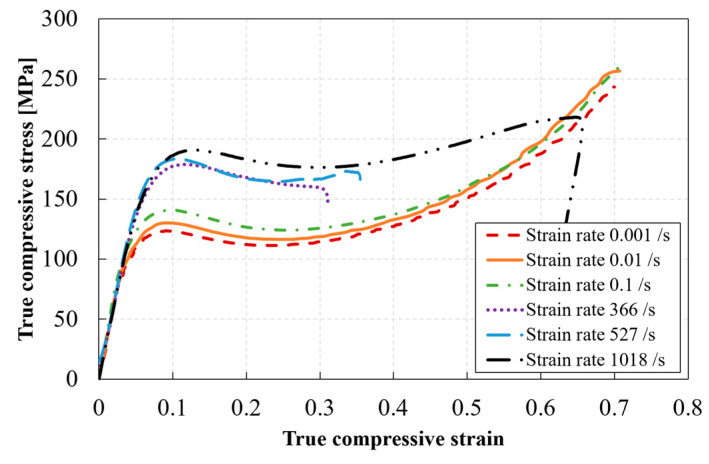
Example of the compressive true stress–true strain curves at different strain rates for the RTM6 epoxy.

Figure 14 shows the true stress–true strain responses of RTM6 epoxy at different strain rates based on measurements from the LVDT and classical Hopkinson analysis. The purpose here is to show the complete response of the material also at higher levels of strains. The indicated strain rates correspond to the strain rate at the yielding stage (calculated in the second region, see Section 3.1). At least three experiments were performed for each testing condition. The curves shown are representative curves for each testing condition. It can be seen that the compressive behavior of the tested epoxy is strain rate sensitive. The true stress–true strain response for all materials follows four different regions: (1) an initial region corresponding to the material’s elastic and viscoelastic behaviors, (2) a non-linear region corresponding to the yielding of the material, which reaches a maximum value at the peak yield point, (3) a strain-softening region after yielding, and (4) further strain hardening until fracture for the quasi-static strain tests, or unloading for the high strain rate tests. The tested epoxy showed an increase in strength with increasing strain rates. Both Gerlach et al. [9] and Morelle et al. [10] reported similar trends for the RTM6 neat resin. Table 2 summarizes the results of the compression tests at different strain rates, based on DIC measurements. Given the achievement of an early quasi-static force equilibrium, combined with the minimization of errors related to interfacial contact conditions, it was possible to calculate the elastic constants at the early stages of loading. The elastic modulus was calculated as the slope of the line on the true stress–true strain curve drawn in the true axial strain range of 0 to 0.02. The Poisson’s ratio was calculated as the slope of the line on the circumferential strain–axial strain curve, see Equation (9), in the same strain range.

### 3.5. Effect of Strain Rate on the Elastic Modulus and Poisson’s Ratio of RTM6 Epoxy in Compression

**Figure 15:**
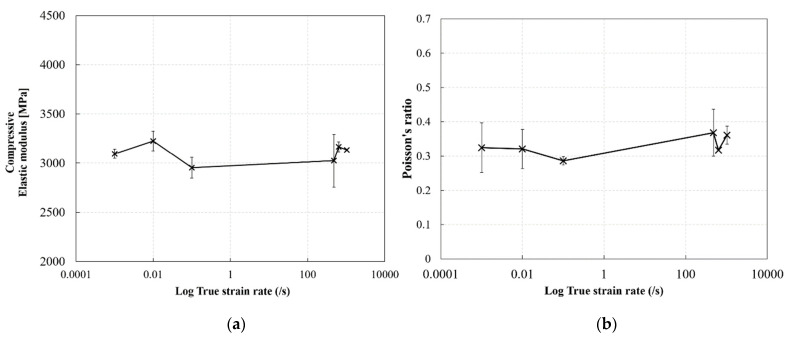
Effect of strain rate on the elastic constants of the RTM6 epoxy: (**a**) elastic modulus, (**b**) Poisson’s ratio.

Figure 15 shows the effect of strain rate on the elastic modulus and Poisson’s ratio of the RTM6 epoxy resin. The compressive elastic modulus and Poisson’s ratio generally showed a constant behavior with increasing strain rate, that is, the differences are within the experimental scatter. The average value of compressive elastic modulus was approx. 3200 MPa, whereas the Poisson’s ratio was approx. 0.32. Similar results were reported by Morelle et al. [10] for an RTM6 epoxy at quasi-static strain rates, and by Fard et al. [4] for a similar epoxy system. Gerlach et al. [4] reported an increase in stiffness with the increase in strain rate for the RTM6 resin, which seems to contradict the current findings. This could be attributed to the fact that the strain rate range covered in the current study was more limited compared to the range in Gerlach’s work. Additionally, and as mentioned earlier, the DMA results revealed that the highly crosslinked RTM6 epoxy resin showed predominantly elastic behavior rather than viscoelastic behavior at the early stages of loading or at small strains. As a consequence, the resin material was expected to be independent of the strain rate at the initial elastic range due to the very small contribution of the damping component in the deformation at high strain rates.

### 3.6. Effect of Strain Rate on the Peak Yield Strength of the RTM6 Epoxy Resin

**Figure 16:**
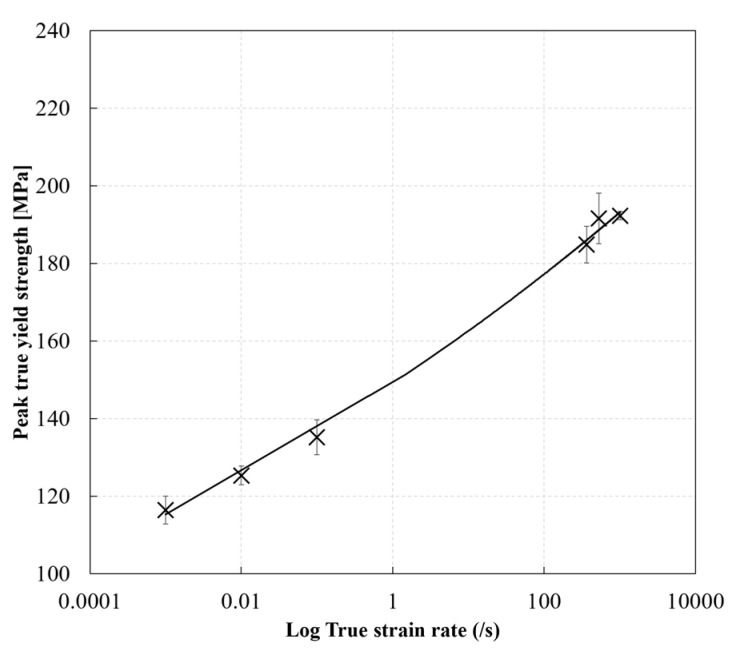
Effect of strain rate on the compressive peak yield strength of the RTM6 epoxy.

Figure 16 shows the effect of strain rate on the true peak yield strength of the RTM6 epoxy resin. The true peak yield strength showed an increase with increasing strain rate. The true peak yield strength increased from approx. 116 MPa at a strain rate of 0.001 s^−1^ to approx. 192 MPa at a strain rate of 1018 s^−1^. This corresponds to a percentage increase of approx. 65%. Similar results were obtained by Gerlach et al. [9] for the RTM6 epoxy, and Rio et al. [21] for a similar epoxy resin. At low strain rates, the viscoelastic resin has enough time to deform. However, at high strain rates, the viscoelastic resin does not have enough time to fully deform due to the reduced molecular mobility of the polymer chains, as reported by Chen et al. [26]. The evolution of the true peak yield strength of the RTM6 epoxy with strain rate can be described by a least square power fit relation with R^2^ values greater than 0.9:

(12) $$\sigma_{t}^{Peak\;yield}=A{\dot{\varepsilon }}^{b}$$

where $\sigma_{t}^{Peak\;yield}$ is the true peak yield strength in compression, *A* is the compressive strength coefficient with a value of 149.19 MPa, and *b* is the strain rate sensitivity exponent 0.0374.

## 4. Conclusions

Several tests were carried out to study the effect of strain rate on the compressive behavior of RTM6 epoxy resin in the strain rate range of 0.001 to 1035 s^−1^. Special attention was paid to the optimization of the test methodologies in order to obtain compression data for the epoxy free of bias related to the use of different load introduction techniques and sample geometries over the considered strain rate range. The sample geometry was carefully selected and appropriate measures were taken to reduce interfacial friction and, thus, sample barreling. A novel self-alignment tool was used to provide better dynamic load introduction and to guarantee perfect interfacial contact. The 3D DIC technique was used to measure the instantaneous full field deformation of the entire sample during compression at different strain rates. Based on the DIC results, the sample barreling and the homogeneity of the axial strain fields were analyzed. Additionally, the use of 3D DIC allowed the calculation of the volume change of the samples during testing. The effect of strain rate on the elastic modulus, the Poisson’s ratio, and the compressive peak yield strength were investigated. It was found that the RTM6 epoxy resin was strain rate sensitive in compression. The compressive peak yield strength showed a significant increase with increasing strain rate. However, the elastic modulus, Poisson’s ratio, and the volume reduction in compression were strain rate independent. Additionally, the 3D DIC technique proved to be very beneficial in extending the capabilities of the compression tests at high strain rates using the split Hopkinson techniques and revealed important testing and material information that was normally assumed. The barreling of the sample in compression, quantified by the barreling ratio, showed an increase during the progression of the compression tests. However, the barreling ratio significantly decreased with increasing strain rate. Moreover, the assumption of conservation of volume in the yielding stage cannot be considered valid. Assuming volume conservation gives rise to a non-negligible underestimation of the strength of the material.

## Figures and Tables

**Table 1:** Processing parameters for DIC.

Parameter	Value
Correlation criterion	Zero normalized sum of square differences (ZNSSD)
Interpolation order	Bi-cubic spline
Shape function	Affine
Subset size (pixels)	55 × 55 (quasi-static) and 21 × 21 (high strain rate)
Step size (pixels)	10
Strain window	15

**Table 2:** Summary of the results of the RMT6 epoxy resin during compression at different strain rates.

Achieved Strain Rates for Elastic Modulus and Poisson’s Ratio (s^−1^)	Elastic Modulus (MPa)		Poisson’s Ratio		Achieved Strain Rates for Peak Yield Strength (s^−1^)	True Peak Yield Strength (MPa)	
	Mean	Std. dev.	Mean	Std. dev.		Mean	Std. dev.
0.001	3242.733	256.889	0.3243	0.1256	0.001	116.403	6.2101
0.01	3358.254	91.116	0.3208	0.0985	0.01	125.318	4.2153
0.1	3250.859	314.344	0.2861	0.0178	0.1	135.196	7.8117
478.638	3293.361	185.774	0.3683	0.09698	365.760	184.840	8.2448
638.877	3307.398	251.415	0.3174	0.0017	527.334	191.603	11.305
1034.972	3270.952	182.501	0.3608	0.0375	1017.751	192.295	1.774

## Data Availability

Not applicable.

## References

[1] Hsieh T.H., Kinloch A.J., Masania K., Taylor A.C., Sprenger S. (2010). The mechanisms and mechanics of the toughening of epoxy polymers modified with silica nanoparticles. Polymer.

[2] Kruckenberg T.M., Paton R. (1998). Resin Transfer Moulding for Aerospace Structures.

[3] Gómez-del Río T., Rodríguez J. (2012). Compression yielding of epoxy: Strain rate and temperature effect. Mater. Des..

[4] Fard M.Y., Liu Y., Chattopadhyay A. (2011). Characterization of Epoxy Resin Including Strain Rate Effects Using Digital Image Correlation System. J. Aerosp. Eng..

[5] Littell J.D., Ruggeri C.R., Goldberg R.K., Roberts G.D., Arnold W.A., Binienda W.K. (2008). Measurement of Epoxy Resin Tension, Compression, and Shear Stress–Strain Curves over a Wide Range of Strain Rates Using Small Test Specimens. J. Aerosp. Eng..

[6] Bernard C.A., Bahlouli N., George D., Rémond Y., Ahzi S. (2020). Identification of the dynamic behavior of epoxy material at large strain over a wide range of temperatures. Mech. Mater..

[7] Poulain X., Kohlman L.W., Binienda W., Roberts G.D., Goldberg R.K., Benzerga A.A. (2013). Determination of the intrinsic behavior of polymers using digital image correlation combined with video-monitored testing. Int. J. Solids Struct..

[8] Naik N.K., Shankar P.J., Kavala V.R., Ravikumar G., Pothnis J.R., Arya H. (2011). High strain rate mechanical behavior of epoxy under compressive loading: Experimental and modeling studies. Mater. Sci. Eng. A.

[9] Gerlach R., Siviour C.R., Petrinic N., Wiegand J. (2008). Experimental characterisation and constitutive modelling of RTM-6 resin under impact loading. Polymer.

[10] Morelle X.P., Chevalier J., Bailly C., Pardoen T., Lani F. (2017). Mechanical characterization and modeling of the deformation and failure of the highly crosslinked RTM6 epoxy resin. Mech. Time-Depend. Mater..

[11] Jerabek M., Major Z., Lang R.W. (2010). Uniaxial compression testing of polymeric materials. Polym. Test..

[12] Pei P., Pei Z., Tang Z. (2020). Numerical and theoretical analysis of the inertia effects and interfacial friction in SHPB test systems. Materials.

[13] Li P., Siviour C.R., Petrinic N. (2009). The effect of strain rate, specimen geometry and lubrication on responses of aluminium AA2024 in uniaxial compression experiments. Exp. Mech..

[14] Zhong W.Z., Rusinek A., Jankowiak T., Abed F., Bernier R., Sutter G. (2015). Influence of interfacial friction and specimen configuration in Split Hopkinson Pressure Bar system. Tribol. Int..

[15] Gorham D.A. (1991). The effect of specimen dimensions on high strain rate compression measurements of copper. J. Phys. D Appl. Phys..

[16] Siviour C.R. (2017). High strain rate characterization of polymers. AIP Conf. Proc..

[17] Tuninetti V., Gilles G., Péron-Lührs V., Habraken A.M. (2012). Compression Test for Metal Characterization using Digital Image Correlation and Inverse Modeling. Procedia IUTAM.

[18] Chen W.W., Song B. (2013). Split Hopkinson (Kolsky) Bar: Design, Testing and Applications.

[19] Sunny G., Yuan F., Prakash V., Lewandowski J. (2009). Design of inserts for split-hopkinson pressure bar testing of low strain-to-failure materials. Exp. Mech..

[20] Miao Y.G., Li Y.L., Liu H.Y., Deng Q., Shen L., Mai Y.W., Guo Y.Z., Suo T., Hu H.T., Xie F.Q. (2016). Determination of dynamic elastic modulus of polymeric materials using vertical split Hopkinson pressure bar. Int. J. Mech. Sci..

[21] Chen W.W., Rajendran A.M., Song B., Nie X. (2007). Dynamic fracture of ceramics in armor applications. J. Am. Ceram. Soc..

[22] Othman R. (2018). The Kolsky-Hopkinson Bar Machine: Selected Topics.

[23] Zotti A., Elmahdy A., Zuppolini S., Borriello A., Verleysen P., Zarrelli M. (2020). Aromatic Hyperbranched Polyester/RTM6 Epoxy Resin for EXTREME Dynamic Loading Aeronautical Applications. Nanomaterials.

[24] Elmahdy A., Zotti A., Zuppolini S., Zarrelli M., Borriello A., Verleysen P. (2021). Effect of Strain Rate and Silica Filler Content on the Compressive Behavior of RTM6 Epoxy—Based Nanocomposites. Polymers.

[25] Kolsky H. (1949). An Investigation of the Mechanical Properties of Materials at very High Rates of Loading. Proc. Phys. Soc. Sect. B.

[26] Chen L.P., Yee A.F., Moskala E.J. (1999). The Molecular Basis for the Relationship between the Secondary Relaxation and Mechanical Properties of a Series of Polyester Copolymer Glasses. Macromolecules.

